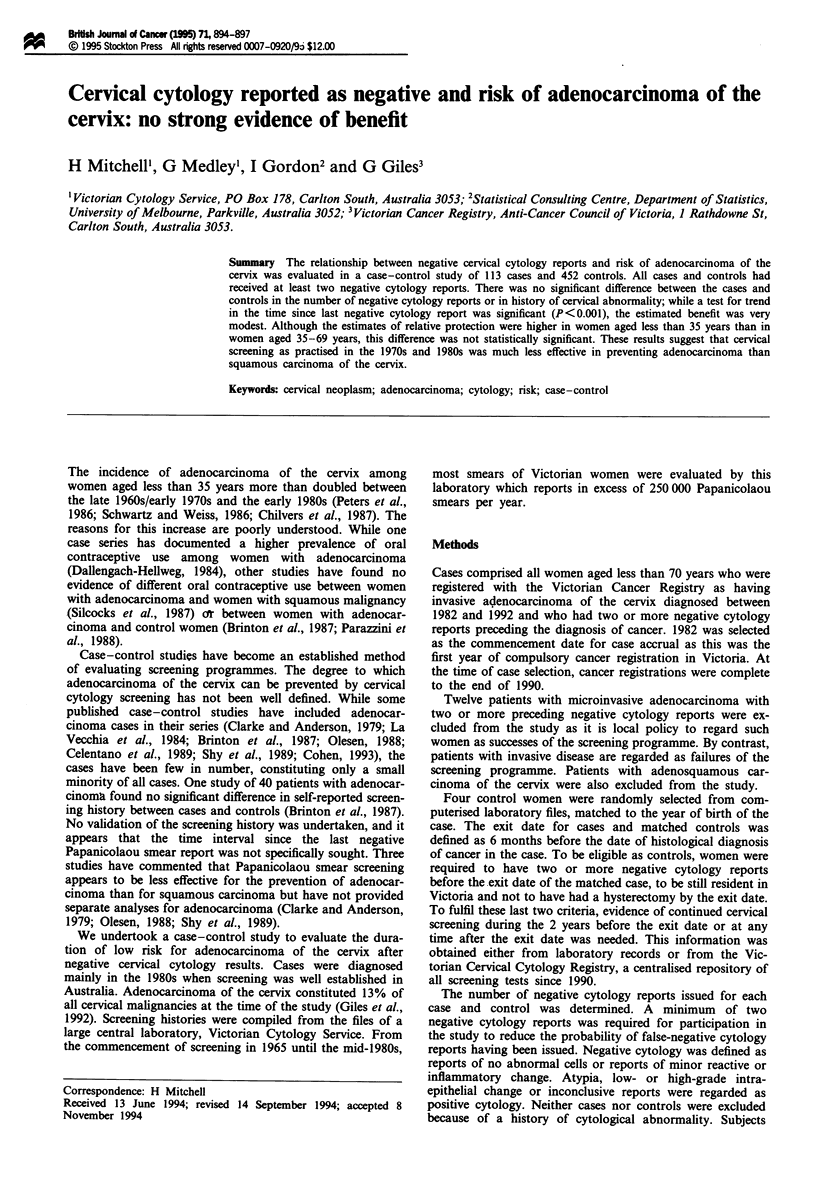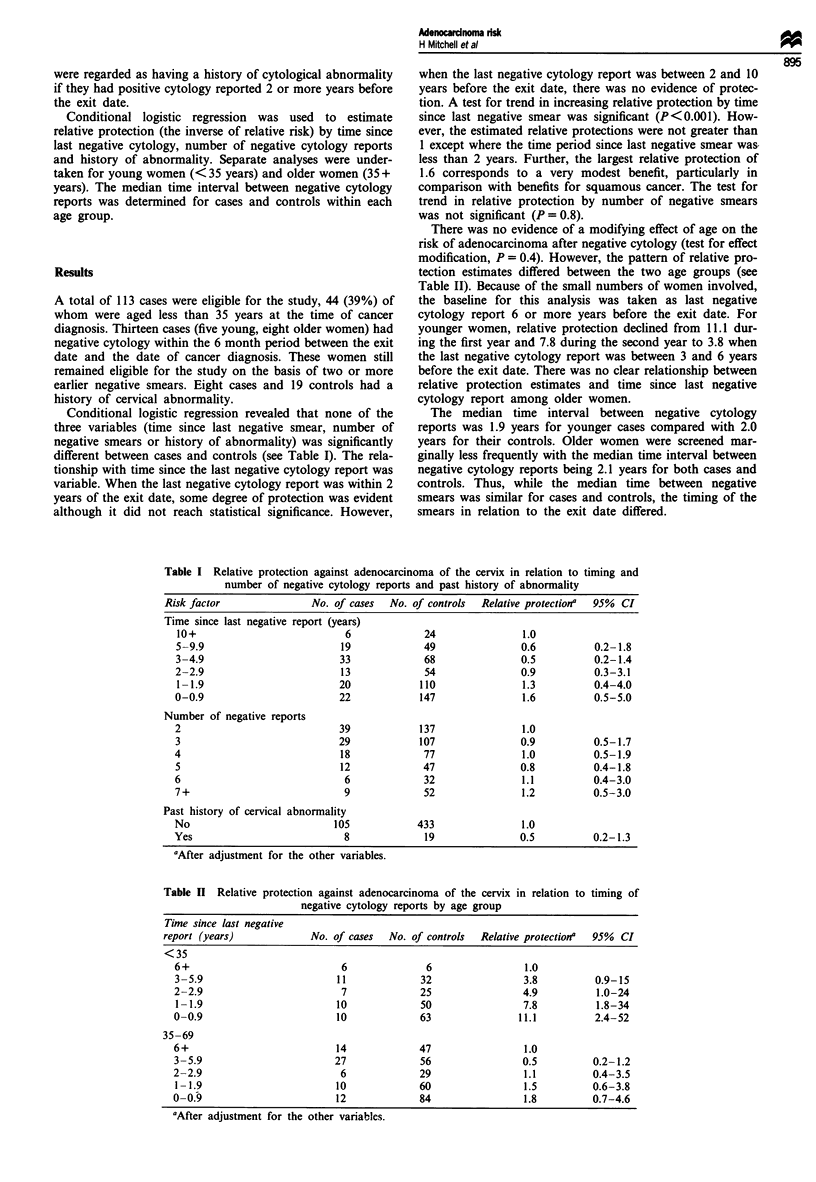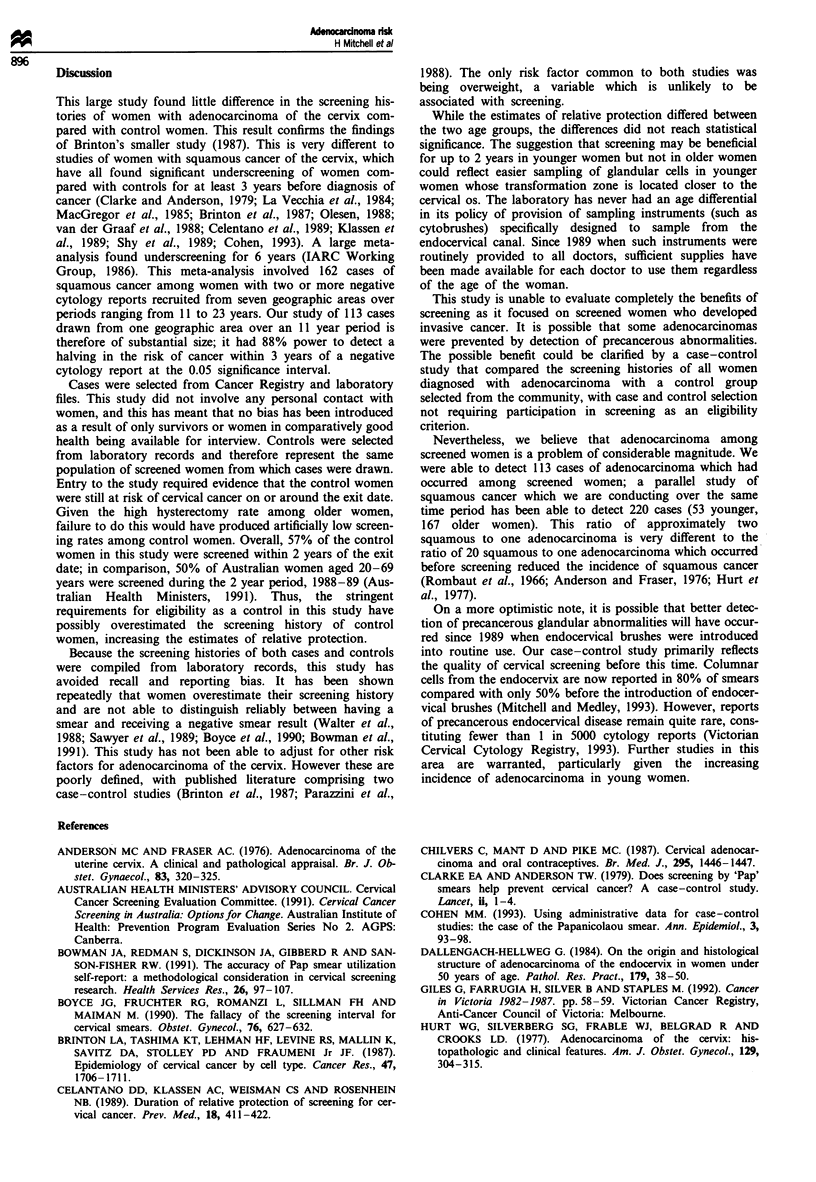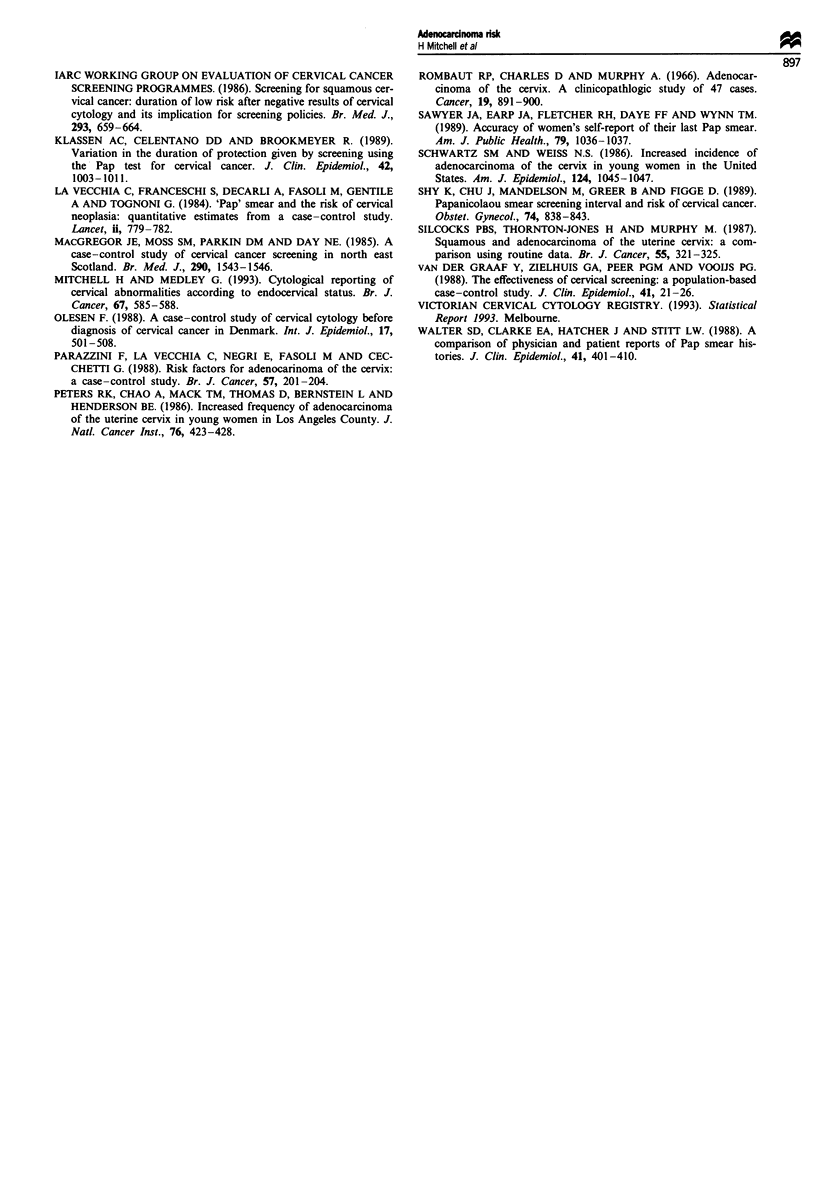# Cervical cytology reported as negative and risk of adenocarcinoma of the cervix: no strong evidence of benefit.

**DOI:** 10.1038/bjc.1995.172

**Published:** 1995-04

**Authors:** H. Mitchell, G. Medley, I. Gordon, G. Giles

**Affiliations:** Victorian Cytology Service, Carlton South, Australia.

## Abstract

The relationship between negative cervical cytology reports and risk of adenocarcinoma of the cervix was evaluated in a case-control study of 113 cases and 452 controls. All cases and controls had received at least two negative cytology reports. There was no significant difference between the cases and controls in the number of negative cytology reports or in history of cervical abnormality; while a test for trend in the time since last negative cytology report was significant (P < 0.001), the estimated benefit was very modest. Although the estimates of relative protection were higher in women aged less than 35 years than in women aged 35-69 years, this difference was not statistically significant. These results suggest that cervical screening as practised in the 1970s and 1980s was much less effective in preventing adenocarcinoma than squamous carcinoma of the cervix.


British Journal of Cancer (1995) 71, 894-897

© 1995 Stockton Press All rights reserved 0007-0920/95 $12.00

### Cervical cytology reported as negative and risk of adenocarcinoma of the cervix: no strong evidence of benefit

H Mitchell[1], G Medley[1], I Gordon[2] and G Giles[3]

[1]*Victorian Cytology Service, PO Box 178, Carlton South, Australia 3053;* [2]*Statistical Consulting Centre, Department of Statistics, University of Melbourne, Parkville, Australia 3052;* [3]*Victorian Cancer Registry, Anti-Cancer Council of Victoria, 1 Rathdowne St, Carlton South, Australia 3053.*

**Summary** The relationship between negative cervical cytology reports and risk of adenocarcinoma of the cervix was evaluated in a case-control study of 113 cases and 452 controls. All cases and controls had received at least two negative cytology reports. There was no significant difference between the cases and controls in the number of negative cytology reports or in history of cervical abnormality; while a test for trend in the time since last negative cytology report was significant ($P<0.001$), the estimated benefit was very modest. Although the estimates of relative protection were higher in women aged less than 35 years than in women aged 35-69 years, this difference was not statistically significant. These results suggest that cervical screening as practised in the 1970s and 1980s was much less effective in preventing adenocarcinoma than squamous carcinoma of the cervix.

**Keywords:** cervical neoplasm; adenocarcinoma; cytology; risk; case-control

The incidence of adenocarcinoma of the cervix among women aged less than 35 years more than doubled between the late 1960s/early 1970s and the early 1980s (Peters *et al.*, 1986; Schwartz and Weiss, 1986; Chilvers *et al.*, 1987). The reasons for this increase are poorly understood. While one case series has documented a higher prevalence of oral contraceptive use among women with adenocarcinoma (Dallengach-Hellweg, 1984), other studies have found no evidence of different oral contraceptive use between women with adenocarcinoma and women with squamous malignancy (Silcocks *et al.*, 1987) or between women with adenocarcinoma and control women (Brinton *et al.*, 1987; Parazzini *et al.*, 1988).

Case-control studies have become an established method of evaluating screening programmes. The degree to which adenocarcinoma of the cervix can be prevented by cervical cytology screening has not been well defined. While some published case-control studies have included adenocarcinoma cases in their series (Clarke and Anderson, 1979; La Vecchia *et al.*, 1984; Brinton *et al.*, 1987; Olesen, 1988; Celentano *et al.*, 1989; Shy *et al.*, 1989; Cohen, 1993), the cases have been few in number, constituting only a small minority of all cases. One study of 40 patients with adenocarcinoma found no significant difference in self-reported screening history between cases and controls (Brinton *et al.*, 1987). No validation of the screening history was undertaken, and it appears that the time interval since the last negative Papanicolaou smear report was not specifically sought. Three studies have commented that Papanicolaou smear screening appears to be less effective for the prevention of adenocarcinoma than for squamous carcinoma but have not provided separate analyses for adenocarcinoma (Clarke and Anderson, 1979; Olesen, 1988; Shy *et al.*, 1989).

We undertook a case-control study to evaluate the duration of low risk for adenocarcinoma of the cervix after negative cervical cytology results. Cases were diagnosed mainly in the 1980s when screening was well established in Australia. Adenocarcinoma of the cervix constituted 13% of all cervical malignancies at the time of the study (Giles *et al.*, 1992). Screening histories were compiled from the files of a large central laboratory, Victorian Cytology Service. From the commencement of screening in 1965 until the mid-1980s, most smears of Victorian women were evaluated by this laboratory which reports in excess of 250 000 Papanicolaou smears per year.

Correspondence: H Mitchell
Received 13 June 1994; revised 14 September 1994; accepted 8 November 1994

### Methods

Cases comprised all women aged less than 70 years who were registered with the Victorian Cancer Registry as having invasive adenocarcinoma of the cervix diagnosed between 1982 and 1992 and who had two or more negative cytology reports preceding the diagnosis of cancer. 1982 was selected as the commencement date for case accrual as this was the first year of compulsory cancer registration in Victoria. At the time of case selection, cancer registrations were complete to the end of 1990.

Twelve patients with microinvasive adenocarcinoma with two or more preceding negative cytology reports were excluded from the study as it is local policy to regard such women as successes of the screening programme. By contrast, patients with invasive disease are regarded as failures of the screening programme. Patients with adenosquamous carcinoma of the cervix were also excluded from the study.

Four control women were randomly selected from computerised laboratory files, matched to the year of birth of the case. The exit date for cases and matched controls was defined as 6 months before the date of histological diagnosis of cancer in the case. To be eligible as controls, women were required to have two or more negative cytology reports before the exit date of the matched case, to be still resident in Victoria and not to have had a hysterectomy by the exit date. To fulfil these last two criteria, evidence of continued cervical screening during the 2 years before the exit date or at any time after the exit date was needed. This information was obtained either from laboratory records or from the Victorian Cervical Cytology Registry, a centralised repository of all screening tests since 1990.

The number of negative cytology reports issued for each case and control was determined. A minimum of two negative cytology reports was required for participation in the study to reduce the probability of false-negative cytology reports having been issued. Negative cytology was defined as reports of no abnormal cells or reports of minor reactive or inflammatory change. Atypia, low- or high-grade intraepithelial change or inconclusive reports were regarded as positive cytology. Neither cases nor controls were excluded because of a history of cytological abnormality. Subjects were regarded as having a history of cytological abnormality if they had positive cytology reported 2 or more years before the exit date.

Conditional logistic regression was used to estimate relative protection (the inverse of relative risk) by time since last negative cytology, number of negative cytology reports and history of abnormality. Separate analyses were undertaken for young women (<35 years) and older women (35+ years). The median time interval between negative cytology reports was determined for cases and controls within each age group.

### Results

A total of 113 cases were eligible for the study, 44 (39%) of whom were aged less than 35 years at the time of cancer diagnosis. Thirteen cases (five young, eight older women) had negative cytology within the 6 month period between the exit date and the date of cancer diagnosis. These women still remained eligible for the study on the basis of two or more earlier negative smears. Eight cases and 19 controls had a history of cervical abnormality.

Conditional logistic regression revealed that none of the three variables (time since last negative smear, number of negative smears or history of abnormality) was significantly different between cases and controls (see Table I). The relationship with time since the last negative cytology report was variable. When the last negative cytology report was within 2 years of the exit date, some degree of protection was evident although it did not reach statistical significance. However, when the last negative cytology report was between 2 and 10 years before the exit date, there was no evidence of protection. A test for trend in increasing relative protection by time since last negative smear was significant ($P<0.001$). However, the estimated relative protections were not greater than 1 except where the time period since last negative smear was less than 2 years. Further, the largest relative protection of 1.6 corresponds to a very modest benefit, particularly in comparison with benefits for squamous cancer. The test for trend in relative protection by number of negative smears was not significant ($P=0.8$).

There was no evidence of a modifying effect of age on the risk of adenocarcinoma after negative cytology (test for effect modification, $P=0.4$). However, the pattern of relative protection estimates differed between the two age groups (see Table II). Because of the small numbers of women involved, the baseline for this analysis was taken as last negative cytology report 6 or more years before the exit date. For younger women, relative protection declined from 11.1 during the first year and 7.8 during the second year to 3.8 when the last negative cytology report was between 3 and 6 years before the exit date. There was no clear relationship between relative protection estimates and time since last negative cytology report among older women.

The median time interval between negative cytology reports was 1.9 years for younger cases compared with 2.0 years for their controls. Older women were screened marginally less frequently with the median time interval between negative cytology reports being 2.1 years for both cases and controls. Thus, while the median time between negative smears was similar for cases and controls, the timing of the smears in relation to the exit date differed.

**Table I** Relative protection against adenocarcinoma of the cervix in relation to timing and number of negative cytology reports and past history of abnormality

| *Risk factor* | *No. of cases* | *No. of controls* | *Relative protection*[a] | *95% CI* |
|---|---|---|---|---|
| Time since last negative report (years) | | | | |
| 10+ | 6 | 24 | 1.0 | |
| 5–9.9 | 19 | 49 | 0.6 | 0.2–1.8 |
| 3–4.9 | 33 | 68 | 0.5 | 0.2–1.4 |
| 2–2.9 | 13 | 54 | 0.9 | 0.3–3.1 |
| 1–1.9 | 20 | 110 | 1.3 | 0.4–4.0 |
| 0–0.9 | 22 | 147 | 1.6 | 0.5–5.0 |
| Number of negative reports | | | | |
| 2 | 39 | 137 | 1.0 | |
| 3 | 29 | 107 | 0.9 | 0.5–1.7 |
| 4 | 18 | 77 | 1.0 | 0.5–1.9 |
| 5 | 12 | 47 | 0.8 | 0.4–1.8 |
| 6 | 6 | 32 | 1.1 | 0.4–3.0 |
| 7+ | 9 | 52 | 1.2 | 0.5–3.0 |
| Past history of cervical abnormality | | | | |
| No | 105 | 433 | 1.0 | |
| Yes | 8 | 19 | 0.5 | 0.2–1.3 |

[a]After adjustment for the other variables.

**Table II** Relative protection against adenocarcinoma of the cervix in relation to timing of negative cytology reports by age group

| *Time since last negative report (years)* | *No. of cases* | *No. of controls* | *Relative protection*[a] | *95% CI* |
|---|---|---|---|---|
| <35 | | | | |
| 6+ | 6 | 6 | 1.0 | |
| 3–5.9 | 11 | 32 | 3.8 | 0.9–15 |
| 2–2.9 | 7 | 25 | 4.9 | 1.0–24 |
| 1–1.9 | 10 | 50 | 7.8 | 1.8–34 |
| 0–0.9 | 10 | 63 | 11.1 | 2.4–52 |
| 35–69 | | | | |
| 6+ | 14 | 47 | 1.0 | |
| 3–5.9 | 27 | 56 | 0.5 | 0.2–1.2 |
| 2–2.9 | 6 | 29 | 1.1 | 0.4–3.5 |
| 1–1.9 | 10 | 60 | 1.5 | 0.6–3.8 |
| 0–0.9 | 12 | 84 | 1.8 | 0.7–4.6 |

[a]After adjustment for the other variables.

### Discussion

This large study found little difference in the screening histories of women with adenocarcinoma of the cervix compared with control women. This result confirms the findings of Brinton's smaller study (1987). This is very different to studies of women with squamous cancer of the cervix, which have all found significant underscreening of women compared with controls for at least 3 years before diagnosis of cancer (Clarke and Anderson, 1979; La Vecchia *et al.*, 1984; MacGregor *et al.*, 1985; Brinton *et al.*, 1987; Olesen, 1988; van der Graaf *et al.*, 1988; Celentano *et al.*, 1989; Klassen *et al.*, 1989; Shy *et al.*, 1989; Cohen, 1993). A large meta-analysis found underscreening for 6 years (IARC Working Group, 1986). This meta-analysis involved 162 cases of squamous cancer among women with two or more negative cytology reports recruited from seven geographic areas over periods ranging from 11 to 23 years. Our study of 113 cases drawn from one geographic area over an 11 year period is therefore of substantial size; it had 88% power to detect a halving in the risk of cancer within 3 years of a negative cytology report at the 0.05 significance interval.

Cases were selected from Cancer Registry and laboratory files. This study did not involve any personal contact with women, and this has meant that no bias has been introduced as a result of only survivors or women in comparatively good health being available for interview. Controls were selected from laboratory records and therefore represent the same population of screened women from which cases were drawn. Entry to the study required evidence that the control women were still at risk of cervical cancer on or around the exit date. Given the high hysterectomy rate among older women, failure to do this would have produced artificially low screening rates among control women. Overall, 57% of the control women in this study were screened within 2 years of the exit date; in comparison, 50% of Australian women aged 20–69 years were screened during the 2 year period, 1988–89 (Australian Health Ministers, 1991). Thus, the stringent requirements for eligibility as a control in this study have possibly overestimated the screening history of control women, increasing the estimates of relative protection.

Because the screening histories of both cases and controls were compiled from laboratory records, this study has avoided recall and reporting bias. It has been shown repeatedly that women overestimate their screening history and are not able to distinguish reliably between having a smear and receiving a negative smear result (Walter *et al.*, 1988; Sawyer *et al.*, 1989; Boyce *et al.*, 1990; Bowman *et al.*, 1991). This study has not been able to adjust for other risk factors for adenocarcinoma of the cervix. However these are poorly defined, with published literature comprising two case–control studies (Brinton *et al.*, 1987; Parazzini *et al.*, 1988). The only risk factor common to both studies was being overweight, a variable which is unlikely to be associated with screening.

While the estimates of relative protection differed between the two age groups, the differences did not reach statistical significance. The suggestion that screening may be beneficial for up to 2 years in younger women but not in older women could reflect easier sampling of glandular cells in younger women whose transformation zone is located closer to the cervical os. The laboratory has never had an age differential in its policy of provision of sampling instruments (such as cytobrushes) specifically designed to sample from the endocervical canal. Since 1989 when such instruments were routinely provided to all doctors, sufficient supplies have been made available for each doctor to use them regardless of the age of the woman.

This study is unable to evaluate completely the benefits of screening as it focused on screened women who developed invasive cancer. It is possible that some adenocarcinomas were prevented by detection of precancerous abnormalities. The possible benefit could be clarified by a case–control study that compared the screening histories of all women diagnosed with adenocarcinoma with a control group selected from the community, with case and control selection not requiring participation in screening as an eligibility criterion.

Nevertheless, we believe that adenocarcinoma among screened women is a problem of considerable magnitude. We were able to detect 113 cases of adenocarcinoma which had occurred among screened women; a parallel study of squamous cancer which we are conducting over the same time period has been able to detect 220 cases (53 younger, 167 older women). This ratio of approximately two squamous to one adenocarcinoma is very different to the ratio of 20 squamous to one adenocarcinoma which occurred before screening reduced the incidence of squamous cancer (Rombaut *et al.*, 1966; Anderson and Fraser, 1976; Hurt *et al.*, 1977).

On a more optimistic note, it is possible that better detection of precancerous glandular abnormalities will have occurred since 1989 when endocervical brushes were introduced into routine use. Our case–control study primarily reflects the quality of cervical screening before this time. Columnar cells from the endocervix are now reported in 80% of smears compared with only 50% before the introduction of endocervical brushes (Mitchell and Medley, 1993). However, reports of precancerous endocervical disease remain quite rare, constituting fewer than 1 in 5000 cytology reports (Victorian Cervical Cytology Registry, 1993). Further studies in this area are warranted, particularly given the increasing incidence of adenocarcinoma in young women.

### References

ANDERSON MC AND FRASER AC. (1976). Adenocarcinoma of the uterine cervix. A clinical and pathological appraisal. *Br. J. Obstet. Gynaecol.*, **83**, 320–325.

AUSTRALIAN HEALTH MINISTERS' ADVISORY COUNCIL. Cervical Cancer Screening Evaluation Committee. (1991). *Cervical Cancer Screening in Australia: Options for Change*. Australian Institute of Health: Prevention Program Evaluation Series No 2. AGPS: Canberra.

BOWMAN JA, REDMAN S, DICKINSON JA, GIBBERD R AND SANSON-FISHER RW. (1991). The accuracy of Pap smear utilization self-report: a methodological consideration in cervical screening research. *Health Services Res.*, **26**, 97–107.

BOYCE JG, FRUCHTER RG, ROMANZI L, SILLMAN FH AND MAIMAN M. (1990). The fallacy of the screening interval for cervical smears. *Obstet. Gynecol.*, **76**, 627–632.

BRINTON LA, TASHIMA KT, LEHMAN HF, LEVINE RS, MALLIN K, SAVITZ DA, STOLLEY PD AND FRAUMENI Jr JF. (1987). Epidemiology of cervical cancer by cell type. *Cancer Res.*, **47**, 1706–1711.

CELANTANO DD, KLASSEN AC, WEISMAN CS AND ROSENHEIN NB. (1989). Duration of relative protection of screening for cervical cancer. *Prev. Med.*, **18**, 411–422.

CHILVERS C, MANT D AND PIKE MC. (1987). Cervical adenocarcinoma and oral contraceptives. *Br. Med. J.*, **295**, 1446–1447.

CLARKE EA AND ANDERSON TW. (1979). Does screening by 'Pap' smears help prevent cervical cancer? A case–control study. *Lancet*, **ii**, 1–4.

COHEN MM. (1993). Using administrative data for case–control studies: the case of the Papanicolaou smear. *Ann. Epidemiol.*, **3**, 93–98.

DALLENGACH-HELLWEG G. (1984). On the origin and histological structure of adenocarcinoma of the endocervix in women under 50 years of age. *Pathol. Res. Pract.*, **179**, 38–50.

GILES G, FARRUGIA H, SILVER B AND STAPLES M. (1992). *Cancer in Victoria 1982–1987*. pp. 58–59. Victorian Cancer Registry, Anti-Cancer Council of Victoria: Melbourne.

HURT WG, SILVERBERG SG, FRABLE WJ, BELGRAD R AND CROOKS LD. (1977). Adenocarcinoma of the cervix: histopathologic and clinical features. *Am. J. Obstet. Gynecol.*, **129**, 304–315.

IARC WORKING GROUP ON EVALUATION OF CERVICAL CANCER SCREENING PROGRAMMES. (1986). Screening for squamous cervical cancer: duration of low risk after negative results of cervical cytology and its implication for screening policies. *Br. Med. J.*, **293**, 659-664.

KLASSEN AC, CELENTANO DD AND BROOKMEYER R. (1989). Variation in the duration of protection given by screening using the Pap test for cervical cancer. *J. Clin. Epidemiol.*, **42**, 1003-1011.

LA VECCHIA C, FRANCESCHI S, DECARLI A, FASOLI M, GENTILE A AND TOGNONI G. (1984). 'Pap' smear and the risk of cervical neoplasia: quantitative estimates from a case-control study. *Lancet*, **ii**, 779-782.

MACGREGOR JE, MOSS SM, PARKIN DM AND DAY NE. (1985). A case-control study of cervical cancer screening in north east Scotland. *Br. Med. J.*, **290**, 1543-1546.

MITCHELL H AND MEDLEY G. (1993). Cytological reporting of cervical abnormalities according to endocervical status. *Br. J. Cancer*, **67**, 585-588.

OLESEN F. (1988). A case-control study of cervical cytology before diagnosis of cervical cancer in Denmark. *Int. J. Epidemiol.*, **17**, 501-508.

PARAZZINI F, LA VECCHIA C, NEGRI E, FASOLI M AND CECCHETTI G. (1988). Risk factors for adenocarinoma of the cervix: a case-control study. *Br. J. Cancer*, **57**, 201-204.

PETERS RK, CHAO A, MACK TM, THOMAS D, BERNSTEIN L AND HENDERSON BE. (1986). Increased frequency of adenocarcinoma of the uterine cervix in young women in Los Angeles County. *J. Natl. Cancer Inst.*, **76**, 423-428.

ROMBAUT RP, CHARLES D AND MURPHY A. (1966). Adenocarcinoma of the cervix. A clinicopathlogic study of 47 cases. *Cancer*, **19**, 891-900.

SAWYER JA, EARP JA, FLETCHER RH, DAYE FF AND WYNN TM. (1989). Accuracy of women's self-report of their last Pap smear. *Am. J. Public Health.*, **79**, 1036-1037.

SCHWARTZ SM AND WEISS N.S. (1986). Increased incidence of adenocarcinoma of the cervix in young women in the United States. *Am. J. Epidemiol.*, **124**, 1045-1047.

SHY K, CHU J, MANDELSON M, GREER B AND FIGGE D. (1989). Papanicolaou smear screening interval and risk of cervical cancer. *Obstet. Gynecol.*, **74**, 838-843.

SILCOCKS PBS, THORNTON-JONES H AND MURPHY M. (1987). Squamous and adenocarcinoma of the uterine cervix: a comparison using routine data. *Br. J. Cancer*, **55**, 321-325.

VAN DER GRAAF Y, ZIELHUIS GA, PEER PGM AND VOOIJS PG. (1988). The effectiveness of cervical screening: a population-based case-control study. *J. Clin. Epidemiol.*, **41**, 21-26.

VICTORIAN CERVICAL CYTOLOGY REGISTRY. (1993). *Statistical Report 1993*. Melbourne.

WALTER SD, CLARKE EA, HATCHER J AND STITT LW. (1988). A comparison of physician and patient reports of Pap smear histories. *J. Clin. Epidemiol.*, **41**, 401-410.